# Self-care interventions and universal health coverage

**DOI:** 10.2471/BLT.23.290927

**Published:** 2023-12-08

**Authors:** Manjulaa Narasimhan, Priya Karna, Olumuyiwa Ojo, Dhammika Perera, Kate Gilmore

**Affiliations:** a Department of Sexual and Reproductive Health and Research, World Health Organization, 20 Avenue Appia, 1211 Geneva, Switzerland.; b World Health Organization Country Office, New Delhi, India.; c World Health Organization Country Office, Abuja, Nigeria.; d MSI Reproductive Choices, London, England.; e Department of International Development, London School of Economics, London, England.

Self-care is not a new concept, but the public health sector has only recently started actively promoting tools that provide greater autonomy and agency to people without formal health training to manage their health for themselves and those in their care (Box 1). Self-care interventions that can be provided as additional options to facility-based care include diagnostics such as pregnancy, coronavirus disease 2019 (COVID-19) or human immunodeficiency virus self-tests; devices to self-monitor blood glucose and/or blood pressure; and drugs such as emergency contraception or for self-management of medical abortions.

Box 1 **WHO definitions of self-care and self-care interventions**

**What is self-care?**

WHO defines self-care as the ability of individuals, families and communities to promote health, prevent disease, maintain health and cope with illness and disability with or without the support of a health worker.

**What are self-care interventions?**

WHO defines self-care interventions as tools that support self-care. Self-care interventions include evidence-based, quality drugs, devices, diagnostics and/or digital technologies which can be provided fully or partially outside of formal health services and can be used with or without the support of a health worker.

WHO: World Health Organization.Data source: WHO, 2022.^1^

The World Health Organization (WHO) global normative guideline on self-care interventions published in 2019 and revised in 2022^2^ promotes the inclusion into national policies of evidence-based, quality interventions which show positive impact at people and health-systems levels. During the COVID-19 pandemic, when health systems were overstretched and governments had imposed movement restrictions, WHO recommended prioritizing many self-care interventions, including through digital health technologies or over-the-counter access at pharmacies (Fig. 1).^3^ Thus, the COVID-19 pandemic raised awareness among policy-makers of the role that people without formal health training have in managing their health. 

**Fig. 1:**
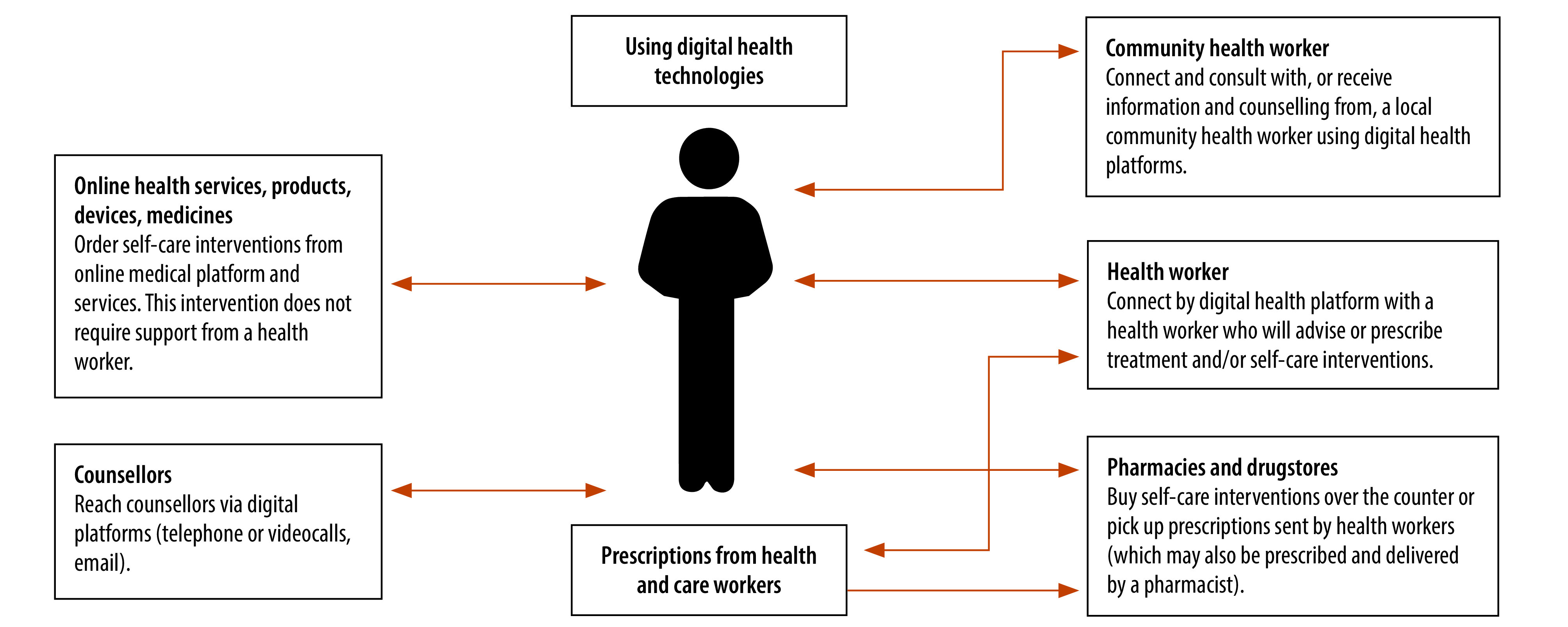
Examples of ways and places of access to self-care interventions

Beyond a pandemic response, the global shortage of health workers needed to achieve and sustain universal health coverage (UHC), necessitates actions that challenge the current modes of how health-care systems operate. Furthermore, identifying effective and feasible policies at national level can improve access to self-care interventions and transform UHC from aspirational to achievable. For example, Nigeria included self-care interventions into the national Task Sharing Standard Operating Procedures. Self-care interventions can meet many health needs, including for quality, reliable, evidence-based and age-appropriate health information; for the availability and accessibility of quality, regulated self-care interventions; and for cost-effective care that does not place clients at financial risk. Several health system challenges often impede the ability of people to access or use health care, resulting in health inequities.^4^ For example, more than half of the world’s population have limited or no access to sexual and reproductive health services over their lifetime. Moreover, by the end of 2022, 108.4 million^5^ people worldwide had been forced to flee their homes because of persecution, conflict, violence and human rights violations. When access to health-care is challenging, self-care can contribute to reducing these inequities.

In this article, we describe how self-care interventions can contribute to reducing inequities by using two examples focused on improving contraceptive needs of women and girls (Fig. 2).

**Fig. 2:**
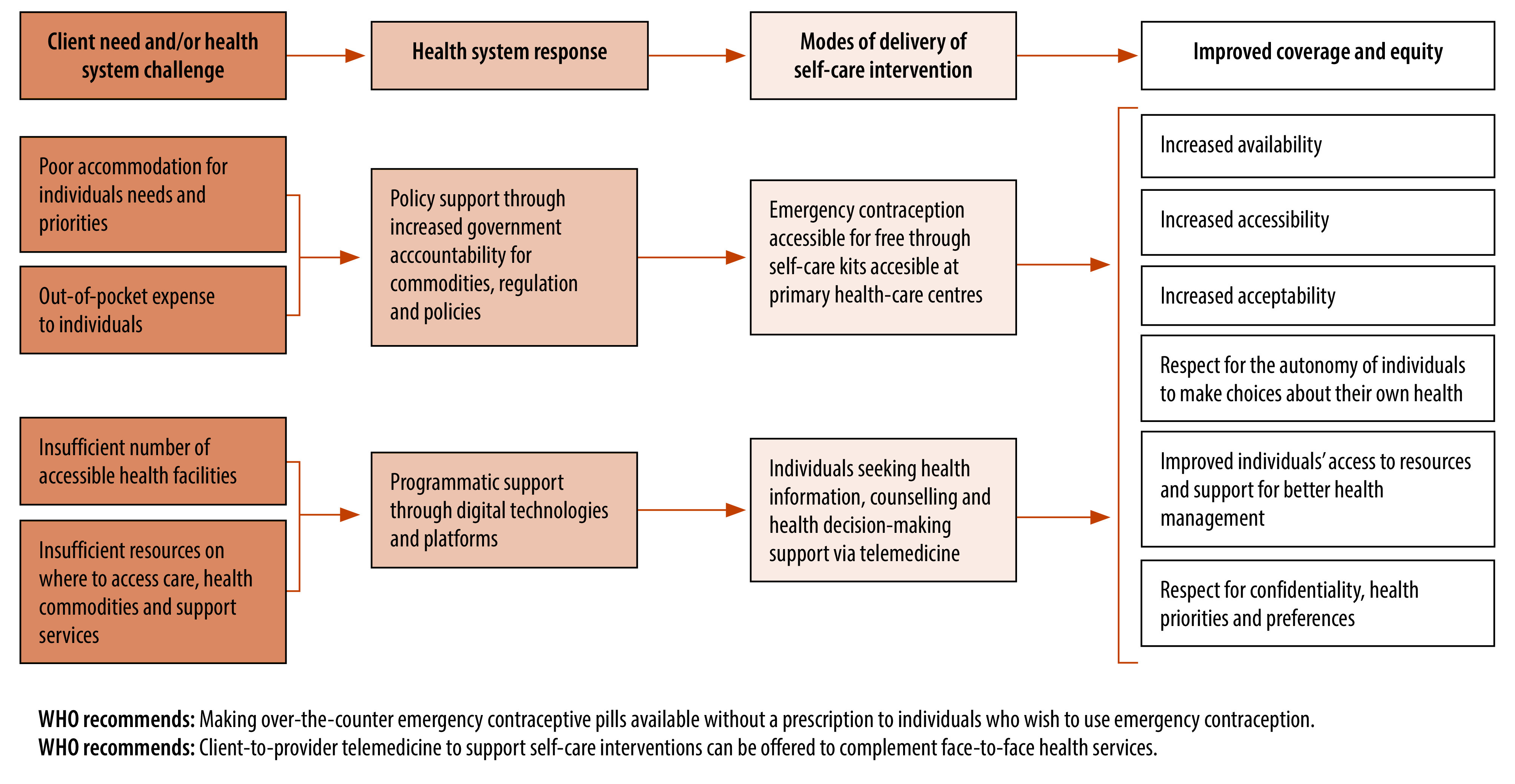
Examples of how self-care interventions can address inequities in health systems

Note: Recommendations are from WHO, 2002.^2^

## Equitable access

Many women access emergency contraception without a prescription over- and/or behind-the-counter, at a pharmacy or drug store.^6^ In many countries, over-the-counter access through pharmacies and other private sector actors comes as an out-of-pocket expenditure to the client. Often referred to as the morning-after pill, use of emergency contraceptive pills is sometimes also marred by stigma, as a marker of moral malaise and irresponsible female sexuality.^7^ Furthermore, some health workers favour prescription-only access and may oppose advance provision, while others may refuse to prescribe it.^8^ As the only method that can be used after unprotected intercourse, emergency contraception is a critical component of the contraceptive methods mix for situations where precoital methods were not used or used incorrectly, a barrier method failed or when sexual assault has taken place, and therefore should be made available for all those who need it.^9^

In April 2023, the Ministry of Health and Family Welfare, Government of India, launched self-care kits, consisting initially of three methods (condoms, emergency contraceptive pills and pregnancy tests), during the second G20 (Group of 20) health working group meeting in Goa, India. The kits were developed with technical support from the WHO Country Office in India. Initially targeting the 150 000 *Ayushman Bharat* health and wellness centres that deliver primary health care free of charge across India, the self-care kits are currently being made available through the public system at all health facilities nationwide. The ministry has also issued a policy directive to all states, with detailed guidelines on how to make self-care kits available free of charge at all public health facilities. The self-care kits are QR-code enabled to provide access to evidence-based information on the use of selected barrier methods and hormonal contraceptives for self-use and safe abortion.

The combination of commodities and information will help to improve contraception access, use and uptake, thereby averting the risk of unwanted pregnancies, increasing early detection of pregnancy, decreasing unsafe abortions and ultimately reducing maternal deaths and morbidities. The kits are also expected to reduce stigmatization of certain individuals and communities at health facilities, including for instance an unmarried adolescent girl who may need access to emergency contraception but fears reaching out to the existing health facility or drug store.

In another example, the international nongovernmental organization MSI Reproductive Choices (MSI) expanded access to self-care interventions in Nigeria to meet the fertility intentions of women and girls through digital health technologies. Based on a model of care that MSI used in Ghana and Mali and other contexts where access to health workers or health facilities was limited,^10^ including during the COVID-19 pandemic,^11^ a telemedicine programme provides women and girls with accurate information and counselling on where to access safe, quality self-care interventions and on which method may best meet their needs, thus enabling them to make informed choices of the most suitable contraceptive methods. MSI set up remote, home-based contact centres and trained contact centre agents who answered calls, including over the phone, WhatsApp and social media. Women could then order the contraceptive option to be delivered by postal service.

However, in Nigeria, the unequal and inequitable access to digital technologies,^12^ the inefficient postal and courier services needed to get commodities to women and for payment and reimbursement of telemedicine visits – including telephone and video visits that increased out-of-pocket expenditure – are all challenges to improved coverage. While programming on self-care interventions should consider these challenges and mitigate their impact on access to quality services, this programme nonetheless showed success in increasing contraceptive coverage and access for women and girls who may have otherwise been at risk of unintended pregnancy. For example, within a 3-month period at the start of the COVID-19 movement restriction policy, over 3000 calls were received, with an overwhelming majority of callers being single and female [personal communication with an MSI representative in Nigeria, April 2022]. Callers were able to interact with a counsellor, enabling the provision of counselling and information on contraceptive choices.

## Conclusion

To make self-care interventions sustainable and equitable, government public health policies must be focused on ensuring that evidence-based, quality self-care options are available and health workers are trained to promote them. Differential health outcomes by race, age, income, class, caste, ethnicity, disability, gender and sexual orientation are the results of duty bearers’ failings, policy-makers’ choices and health providers’ priorities. Low budgets and fragile health systems cannot justify the absence of adequately resourced and effectively implemented public health plans to reach UHC. This gap violates the norms of medical science and of policies enshrined in national laws and in international human rights instruments. Some public policy choices lead to discriminatory health outcomes and they therefore must be challenged.

Increasing momentum for self-care interventions is seen in examples from around the world that show how sound science and innovation in implementing WHO-recommended self-care interventions can meet the needs and rights of clients and patients, as well as the ethical and legal obligations of national policy-makers. While self-care interventions cannot fill all health system gaps and chronic under-resourcing, WHO will continue to expand global evidence-based normative guidance and tools for quality and accessible self-care, enhancing health coverage and advancing the right to health for all. 

The outcomes of the 2023 United Nations high-level meeting on UHC have shown that too many countries are far from reaching the UHC goals, including for sexual and reproductive health. For UHC to be realized, governments need to increase funding and ensure affordable access to health-care facilities. Against global and regional contexts in which Member States’ commitments to health are so unevenly fulfilled, self-care interventions are promising. However, to tackle gaps in health inequities, including for those outside the reach of government-administered health systems, or for those for whom quality, respectful, inclusive health care in health facilities is unattainable, self-care interventions should be implemented within a framework grounded in the key principles of gender equality and human rights.^13^^,^^14^
